# Case Report: Idiopathic pleuroparenchymal fibroelastosis

**DOI:** 10.12688/f1000research.132553.1

**Published:** 2023-08-29

**Authors:** Selsabil Daboussi, Ben Hmida Lenda, Samira Mhamedi, Boubaker Nouha, Chiraz Aichaouia, Aida Ayadi, Zied Moatemri

**Affiliations:** 1 Department of Pneumology, Military Hospital, University of Tunis El Manar, Tunis, 1008, Tunisia; 2 Faculty of Medicine, University of Tunis El Manar, Tunis, 1007, Tunisia; 3 Pathology Department, Abderrahmen Mami Hospital, Tunis, 2080, Tunisia

**Keywords:** elastofibrosis, idiopathic, interstitial lung disease, lung function, pleural thickening, pleuropulmonary elastosis

## Abstract

**Background:** Idiopathic pleuroparenchymal fibroelastosis (IPPFE) is a very rare and a slowly conspicuous progressing chronic lung disease, which usually involves the upper lobes of the lung. This unusual disease, first recognized as a rare idiopathic interstitial pneumonia in 2013, is characterized by dense fibrosis of the visceral pleura and the subjacent lung parenchyma accompanied by elastosis predominating in the subpleural alveolar walls. In the interest of improving our understanding of this uncommon disease, we report a case of IPPFE established by pathology results.

**Case report:** A 73-year-old male patient, smoker, with a medical history of chronic obstructive pulmonary disease, presented since January 2022 with a gradual worsening of dyspnea on exertion and productive cough with weight loss. The chest X-ray detected a thoracic distention. The chest high resolution computed tomography revealed biapical subpleural parenchymatous condensations with tractive bronchiectasis and pleural retraction in the right upper lobe and diffuse bilateral cento-lobular emphysema. A scan-guided trans-parietal lung biopsy showed lung parenchyma tattooed with anthracosic deposits, largely remodeled by fibrous tissue, intermingled with numerous wavy and refractive dyselastotic structures in polarized light. The orcein staining confirmed the presence of excess elastosic fibers within these lesions. All etiological investigations were negative. His lung function studies revealed a reversible obstructive ventilatory disorder. Following a multidisciplinary discussion, the diagnosis of IPPFE was confirmed on the basis of the distribution in the upper lungs on chest computed tomography combined with pathology pattern.

**Conclusions:** This case emphasizes the atypical misleading radiological presentation of IPPFE and the key role of pathological results in establishing the diagnosis. Hence, further studies are needed to improve our understanding of this uncommon disease and to establish clear-cut guidelines for IPPFE diagnosis and management.

**Abbreviations**
COPD: chronic obstructive pulmonary disease
HRCT: High resolution computed tomography
IIP: Idiopathic interstitial pneumonia
IPPFE: Idiopathic pleuroparenchymal fibroelastosis
LABA: Long-acting beta-agonist
LAMA: Long-acting muscarinic antagonist
PPFE: Pleuroparenchymal fibroelastosis
PULF: Pulmonary upper lobe fibrosis

## Introduction

Idiopathic pleuroparenchymal fibroelastosis (IPPFE) is a very rare chronic lung disease, that usually involves the upper lobes of the lungs. ^1^ This unusual disease was first recognized as a rare idiopathic interstitial pneumonia (IIP) in 2013. ^1^ It is characterized by dense fibrosis of the visceral pleura and the subjacent lung parenchyma accompanied by elastosis predominating in the subpleural alveolar walls, with perilobular or bronchocentric distribution. ^2^ It is a slowly conspicuous progressing entity, striking firstly the upper lobes close to the lung apices, producing lung volume loss, leading to platythorax and ultimately an irreversible respiratory failure and early death. ^1^ IPPFE usually presents in adults without gender predilection. ^2^ Although a number of disease associations have been described, its exact cause is still unknown. ^1^ Pleuroparenchymal fibroelastosis (PPFE) is schematically separated into IPPFE and PPFE secondary to a number of conditions. ^3^ Patients often have a history of recurrent pulmonary infections, shortness of breath, and dry cough. ^2^ The diagnosis is based on chest high resolution computed tomography (HRCT), requiring in some cases a pathological confirmation. ^1^ Except for lung transplantation, to this day, there is no specific treatment. ^4^ In the interest of improving our understanding of this uncommon disease, we report a case of IPPFE established by pathology results.

## Case presentation

We represent the case of a 73-year-old male patient, admitted to the Pneumology Department of the Military Hospital of Tunis, Tunisia. He had a medical history of chronic obstructive pulmonary disease (COPD) group E treated with a long-acting beta-agonist (LABA) and long-acting muscarinic antagonist (LAMA). His tobacco consumption amounted to 100 packets per year with no particular exposition to toxics. He had a complete immunization schedule with no history of vaccine reaction or infectious disease. He had no family history of interstitial pneumonia or cancer. In January 2022, he had a gradual worsening of respiratory symptoms, which consisted of productive cough with white sputum and dyspnea on exertion. Recent weight loss was reported. The physical examination had revealed sibilant rales and the saturation at room air was 88%. Cyanosis or digital clubbing were not found. General examination was unremarkable. The laboratory findings were as follows: white blood cells, 10600/L; neutrophils, 5900/L; eosinophil count, 900; hemoglobin, 13.9 g/L; and platelets, 216,000/L. Liver and kidney function was normal. The C-reactive protein was 8 mg/L. Sputum acid-fast bacillus smear, sputum culture and sputum fungal culture test results were all negative. *Aspergillus* antigenemia and serology were also negative. The chest X-ray detected a thoracic distention. He was treated, symptomatically, as an acute exacerbation of COPD.

**Figure 1.**
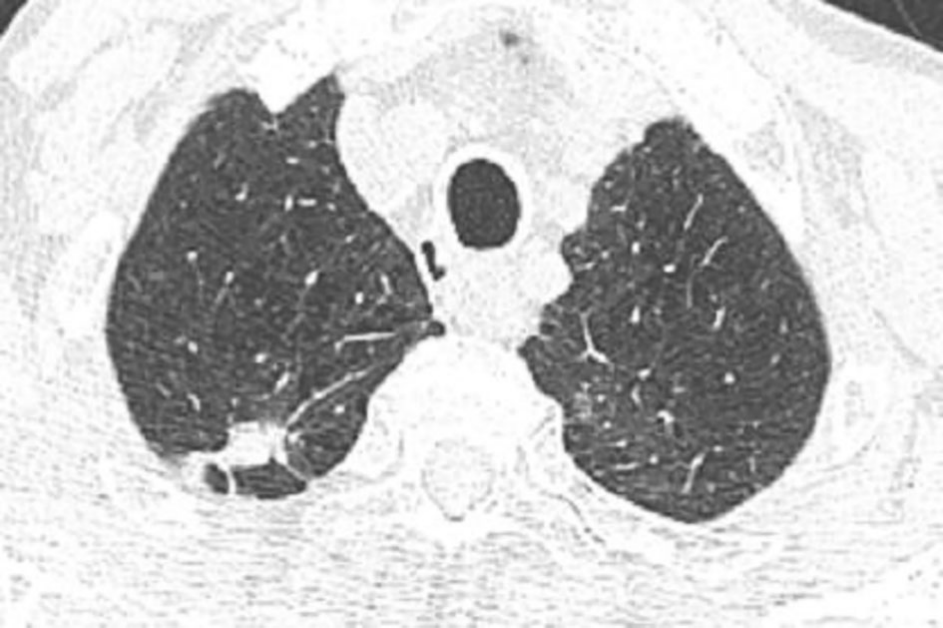
Sub pleural parenchymatous condensations 10*25 mm in the right upper lobe.

**Figure 2.**
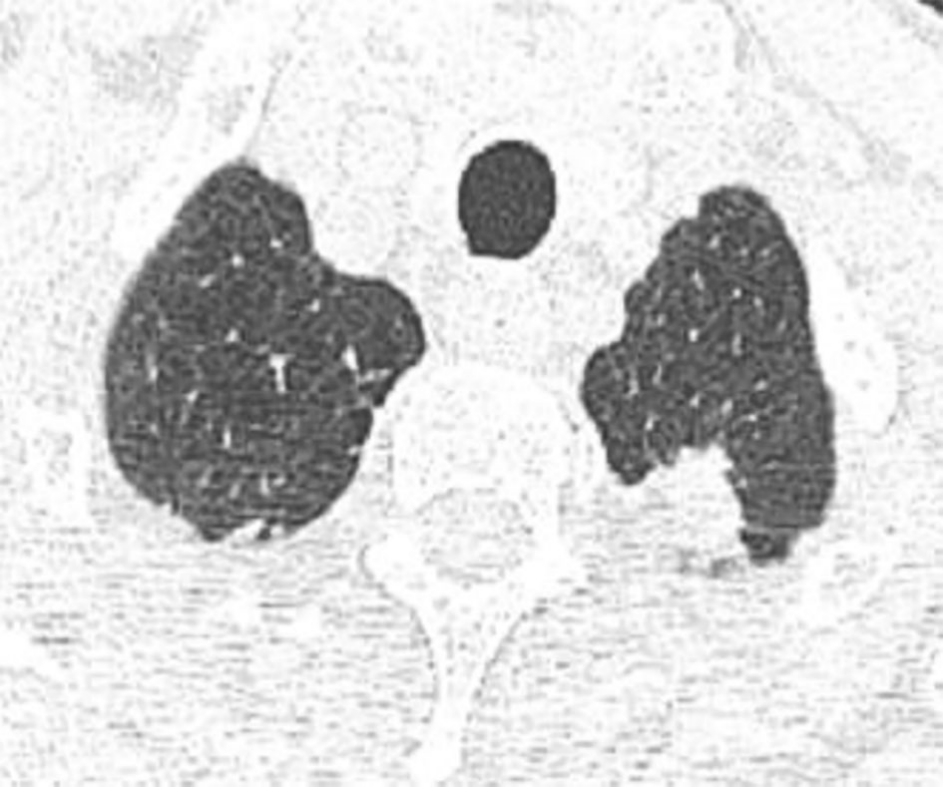
Sub pleural parenchymatous condensations 18*11 mm in the left upper lobe.

Five months after the acute event, a chest HRCT revealed biapical subpleural parenchymatous condensations; 10*25 mm in the right upper lobe ( Figure 1) and 18*11 mm in the left upper lobe ( Figure 2). There was tractive bronchiectasis and pleural retraction in the right upper lobe and diffuse bilateral cento-lobular emphysema.

**Figure 3.**
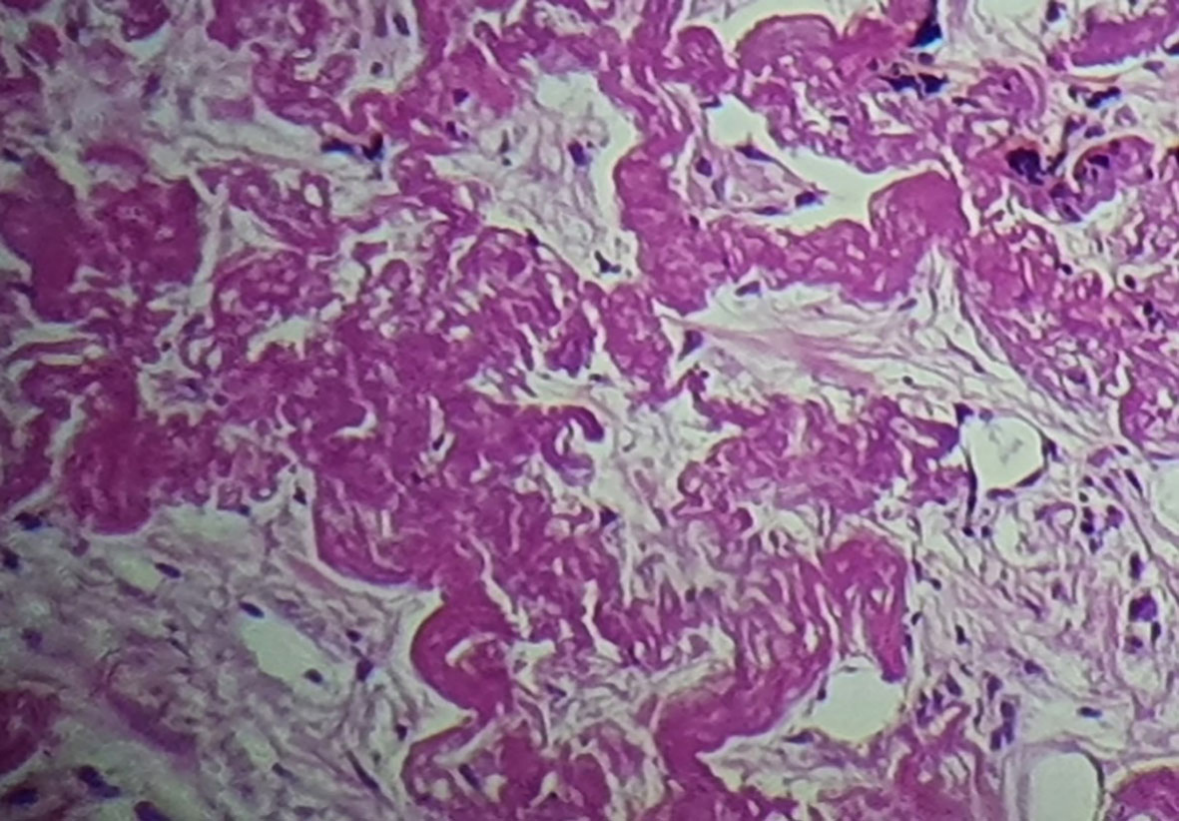
Lung parenchyma tattooed with anthracosic deposits, largely remodeled by fibrous tissue, intermingled with numerous wavy and refractive dyselastotic structures.

**Figure 4.**
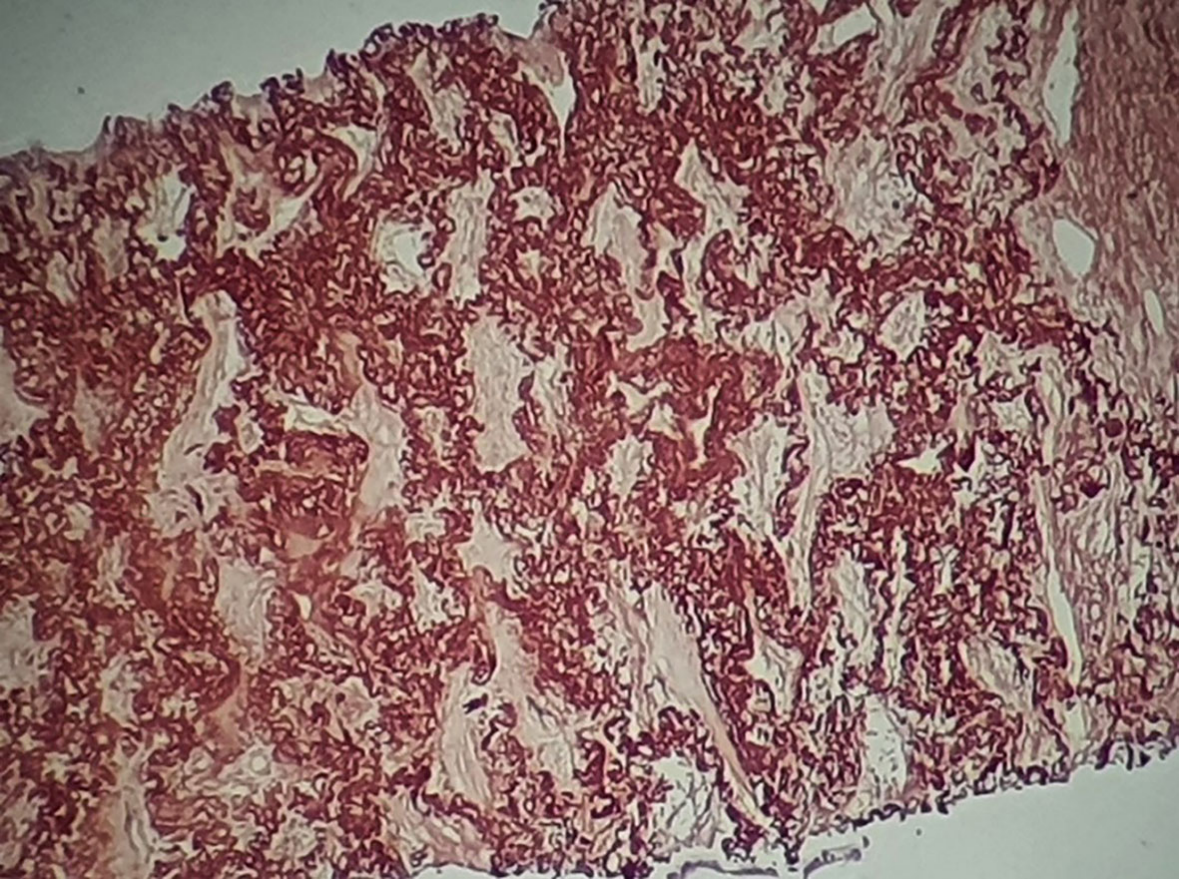
The orcein staining confirms the presence of excess elastotic fibers within these lesions.

A bronchoscopy was performed with unremarkable results. A scan-guided trans-parietal lung biopsy showed lung parenchyma tattooed with anthracotic deposits, largely remodeled by fibrous tissue, intermingled with numerous wavy and refractive dyselastotic structures in polarized light ( Figure 3). The orcein staining confirmed the presence of excess elastotic fibers within these lesions ( Figure 4). Following a multidisciplinary discussion, the diagnosis of IPPFE was confirmed on the basis of the distribution in the upper lungs on chest CT combined with pathology pattern.

His lung function studies revealed a reversible obstructive ventilatory disorder. The results are as follows: forced vital capacity (FVC), 74%; forced expiratory volume in the first second (FEV1), 48%; FEV1/FVC, 50.2% and total lung capacity in a single breath (TLC-SB), 119% **.** He presented with oxygen desaturation at a level of 90% after walking 350 meters during a six-minute walk test.

Serum levels of antinuclear antibodies, rheumatoid factor, anti-cyclic citrulline peptides, antineutrophil cytoplasmic antibodies, extractable nuclear antigen antibodies and dot myositis were all negative.

We recently began treatment with oral corticosteroids, specifically prednisone at a dosage of 40 mg per day, taken once daily.

## Discussion

IPPFE is a rare interstitial pneumonia, characterized by its unique radiological and pathological pattern. ^3^ Historically, IPPFE was acknowledged for at least 20 years as a case of pulmonary upper lobe fibrosis (PULF). ^1^ Only in 2004 was it formally labeled as a PPFE in a mini-series of five patients sharing a distinct radiological and pathological pattern of chronic interstitial and pleural fibrosis of the upper lungs that did not fit within other categories of IIP. ^1^ Most cases were reported in Japan. ^1^ In 2013, IPPFE was first introduced into the classification of IIP as a new category along with idiopathic lymphocytic interstitial pneumonia. ^3^ The true prevalence of IPPFE is still unknown due to the absence of set criteria for its diagnosis. ^5^ Shioya *et al*. detected 29 cases (7.7%) of IPPFE out of 375 cases of IPP over a 10-year period. ^1^

According to a review of 78 cases published up to 2013, IPPFE had a bimodal age distribution ranging between 13 and 85 years of age, with a mean of 49 years; as was reported in our case. ^5^ Although gender predilection is still a controversial topic, a female predominance was found in non-smoking, younger and lightweight patients. ^1^ Unlike our case, indeed, IPPFE mostly occurs in nonsmokers. ^2^

Commonly, clinical presentation is typically characterized by exertional dyspnea, dry cough, weight loss, chest discomfort and recurrent respiratory infections. ^5^ Pneumothorax and pneumomediastinum are less common. ^5^ Our patient, considering his COPD background, had productive cough with white sputum. Clinical exam features include inspiratory crackles on auscultation, platythorax and deepened suprasternal notch; which is contrasting with our case. ^5^

The typic imaging features of PPFE are highly suggestive showing symmetric, bilateral, apical and irregular pleural thickening with few calcifications and bronchiectasis in some cases. ^4^ Middle and lower lungs may be affected in some patients. ^4^ For our patient, chest HRCT showed biapical subpleural parenchymatous condensations, tractive bronchiectasis and pleural retraction in the right upper lobe. In this regard, our case is noteworthy by its atypical radiological presentation suggesting a neoplastic pulmonary disease, considering our patient background.

The main pathological features of PPFE are upper zone pleural fibrosis with subjacent intra-alveolar dense fibrosis and elastosis, keenly dissociated from the adjacent normal parenchyma, with mild focal lymphocyte and plasma cell infiltration on the periphery of the fibrosis. ^4^

In our case, the diagnosis of IPPFE was clearly confirmed based on typical pathology results of a scan-guided trans-parietal lung biopsy.

For a positive diagnosis of PPFE, an agreed consensus statement has yet to be defined. ^2^ Thus, a list of criteria has been proposed in the literature and adopted in clinical practice. ^3^ Reddy *et al*. suggested both radiological and pathological criteria including confidence levels: “definite”, “consistent with” and “inconsistent with” PPFE. ^3^ Considering the unavailability of pathology and the unfavorable risk-effectiveness profile of invasive procedures, Enomoto *et al*. proposed modified criteria requiring progression of disease on imaging rather than pathologic confirmation. ^3^ In all cases, a multidisciplinary discussion is still mandatory for a conclusive diagnosis of IPPFE, as it was reported in our patient. ^2^

PPFE, to this day, has no clear pathogenetic explanation. ^1^ The pathogenesis is thought to involve acute or subacute lung injury, including diffuse alveolar damage with aberrant tissue repair, leading to exuberant interstitial inflammation and subsequent fibrosis. ^1^^,^^5^ The triggering stimuli remains unknown. ^1^

PPFE is schematically separated into IPPFE and PPFE secondary to a number of conditions. ^3^ Given that a clear causative relationship has yet to be established, these conditions are considered more like disease-associated factors or inciting triggers rather than etiologies. ^2^^,^^4^ So far, the strongest association seems to be with a previous organ transplant [lung, bone marrow, hematopoietic stem cell and liver transplantation]. ^1^^,^^2^ The other associated factors are fibrotic interstitial lung disease, recurrent pulmonary infection, autoimmune disease, radiation therapy, chemotherapy, alkylating drugs, environmental exposures to asbestos, silica or aluminum and familial or genetic telomeropathy. ^3^^,^^4^ In our case, our patient was diagnosed with IPPFE after ruling out all these conditions.

This disease prognosis is poor, and most of the patients show disease progression after diagnosis. ^4^ To date, there is no specific medical effective treatment for PPFE, and lung transplantation remains the only therapeutic option. ^4^

## Conclusions

In conclusion, this case emphasizes the importance of improving our understanding of this uncommon disease, presenting an atypical radiological pattern, thus requiring pathology results for establishing the IPPFE diagnosis. Further experiences and studies are needed to better understand the pathogenesis and to establish clear-cut guidelines for PPFE diagnosis and management.

## Consent

Written informed consent for publication of their clinical details and clinical images was obtained from the patient.

## Data Availability

All data underlying the results are available as part of the article and no additional source data are required.
