# Supplementary material for: Case Report: Idiopathic pleuroparenchymal fibroelastosis
Source: F1000Res. 2023 Aug 29;12:1048. [Version 1] doi: 10.12688/f1000research.132553.1 (PMC11255541; doi:10.12688/f1000research.132553.1)
Supplement: Peer review history

# Reviewer report: Reviewer response for version 1

**DOI:** 10.5256/f1000research.145483.r291326

**Published:** 2024-07-17

**Authors:** Claudia Valenzuela

**Affiliations:** 1 Pulmonology Department, Universidad Autónoma de Madrid, Madrid, Spain

This case report describes FEPP, emphasizing its complexity, clinical, radiological and pathological characteristics to establish the diagnosis and the need for specific treatment.

 It comprehensively reviews the main characteristics of the disease, which is informative for clinical practice. Perhaps in the treatment part  the authors could detail more the treatments used in clinical practice and the level of evidence for it.

Are enough details provided of any physical examination and diagnostic tests, treatment given and outcomes?

Yes

Is the case presented with sufficient detail to be useful for other practitioners?

Yes

Is sufficient discussion included of the importance of the findings and their relevance to future understanding of disease processes, diagnosis or treatment?

Yes

Is the background of the case’s history and progression described in sufficient detail?

Yes

Reviewer Expertise:

Interstitial lung diseases

I confirm that I have read this submission and believe that I have an appropriate level of expertise to confirm that it is of an acceptable scientific standard.

# Reviewer report: Reviewer response for version 1

**DOI:** 10.5256/f1000research.145483.r286245

**Published:** 2024-07-01

**Authors:** Dongmei Zhou

**Affiliations:** 1 Xuzhou Medical University,, Xuzhou City, Jiangsu Province,, China

The present article provides a comprehensive overview of the diagnostic process involved in managing a rare disease characterized by its challenging diagnosis and treatment. Insufficient awareness among clinicians regarding this particular condition necessitates the dissemination of this case report, which aims to enhance their understanding and recognition of the disease.

Are enough details provided of any physical examination and diagnostic tests, treatment given and outcomes?

Yes

Is the case presented with sufficient detail to be useful for other practitioners?

Yes

Is sufficient discussion included of the importance of the findings and their relevance to future understanding of disease processes, diagnosis or treatment?

Yes

Is the background of the case’s history and progression described in sufficient detail?

Yes

Reviewer Expertise:

immunogenetics,endocrine system

I confirm that I have read this submission and believe that I have an appropriate level of expertise to confirm that it is of an acceptable scientific standard.
